# Nutritional assessment of nixtamalized maize tortillas produced from dry masa flour, landraces, and high yield hybrids and varieties

**DOI:** 10.3389/fnut.2023.1183935

**Published:** 2023-07-06

**Authors:** Beatriz A. Acosta-Estrada, Sergio O. Serna-Saldívar, Cristina Chuck-Hernández

**Affiliations:** ^1^Tecnológico de Monterrey, School of Engineering and Sciences, Monterrey, Mexico; ^2^Tecnológico de Monterrey, The Institute for Obesity Research, Monterrey, Mexico

**Keywords:** dry masa flour, hybrids, landraces, macronutrients, maize, micronutrients, PDCAAS, tortillas

## Abstract

In the scientific literature there are different analyses of the nutritional profiles of maize tortillas, whether they are landraces or hybrid maize versus those made with dry masa flour (DMF). In general terms, there is agreement in the reported content of moisture. However, for the other nutrients, a great disparity is reported for each type of tortilla which may be due to various factors such as the type of maize or processing methods. In this study, the nutritional aspects of maize tortillas made with different genotypes (five hybrids, two varieties, five landraces, six hybrid mixtures and six dry masa flours) under controlled conditions, were compared. More than 30 characteristics were analyzed. High performance hybrids and varieties (HPHV) and landraces had the highest (*p* < 0.05) antioxidant capacity (58.8% free, 150.2% bound). In terms of vitamins contents, the tortillas produced from DMF contained 11.2 and 3.5 times more B1, 18.6 and 7.8 times more B2, and 2.7 and 5.3 times more B3 than HPHV and landraces respectively; and only in these samples was detected folic acid. DMF tortilla samples contained 1.75 times more sodium and 2.75 times more iron than the other groups, and 0.75 times less calcium than HPHV. Zinc was present in higher concentration (*p* < 0.05) in DMF tortilla samples. The landraces had the highest protein content (average 10.28%), but the tortillas produced from DMF presented the highest protein quality evaluated by protein digestibility-corrected amino acid score (PDCAAS) (*p* < 0.05) that represents 27, 25 and 19% more than hybrids mixture, HPHV and landraces, respectively. This work gives valuable information on how different types of grains differ in the nutritional quality affecting the final product to provide more elements in the decision-making of processors. There is no a perfect maize, but there are genotypes that can be combined as mixtures and the processing method to design superior nutritional tortillas and related products for populations that highly consume them and improve their human health.

## 1. Introduction

Maize (*Zea mays*) plays a crucial role in the livelihoods of millions of people, providing food, feed, and raw material for various industries. Mexico was, in 2021, one of the world’s leading maize producers, with 27 million tons out of the 1.2 billion tons produced worldwide. Maize tortillas, made from masa of nixtamalized corn, are a staple food in Mexico and some countries of Central America. The masa of nixtamalized corn can be either fresh or rehydrated from dry masa flour. The nutritional quality of these tortillas can vary depending on the genotype used and the process techniques in their production. Mexico has diverse genotypes as landraces, varieties, and hybrids across different regions (1). Landraces are diverse traditional open-pollinated varieties adapted to specific regions over generations, while hybrids and varieties, which are more uniform in their genetic makeup and have been developed through modern breeding techniques, aim to produce higher yields. Additionally, varieties are created through selective breeding over at least 7 generations, while hybrids are the result of crossing two genetically uniform inbred lines. With the increasing demand for maize, there has been a shift toward cultivating high-yield hybrids and varieties to meet the demand for food, feed and industry. The nutritional quality of maize tortillas produced from landraces and high-yield hybrids and varieties has been extensively studied. Nonetheless, current studies in this field tend to examine simple proximal factors, phytochemical profile and antioxidant capacity, and sometimes minerals (2–5). However, these studies often fail to consider the complexity of the situation, as most of the landraces being evaluated are blue. Furthermore, these evaluations typically compare landraces to white commercial counterparts without specifying genotype, and the tortillas are mostly purchased, made without their input or control. This raises concerns about the impact of the shift toward high-yield hybrids and varieties on the nutritional quality of maize tortillas, which is the most relevant dietary staple in Mexico.

It is well known that the type of maize variety used in the field affects the crop yield by hectare. For example, Martínez-Gutiérrez et al. (6) reported the yield of white maize hybrids in different locations in the highlands (Valles Altos) of Mexico, highlighting the potential benefits of high-yield varieties for food security and livelihoods. In contrast, Tadeo-Robledo et al. (7) investigated the yield of native and hybrid maize varieties in different planting dates and thermal units, suggesting that the choice of variety and planting time can significantly impact yield productivity and resource use efficiency. Many production decisions are made based on agronomic factors, market factors, and policy, such as higher yields, disease resistance, uniformity of quality, and subsidies for hybrids without considering the nutritional traits of final products. In coming years, attractive options for farmers in Latin America will be those of improvements in higher yields and resistance to climate change stresses (8).

Mexican consumers seek native-traditional foods to improve their intake of nutrients and health-promoting phytochemicals (2). However, there is no information about the overall performance of different maize genotypes on the nutritional traits of tortillas. For example, Salinas Moreno et al. (5), evaluated the antioxidant capacity, phenolic content, and fatty acid profile of different colored maize varieties, finding that some have higher antioxidant and nutraceutical potential than others. Herrera-Sotero et al. (9) investigate the chemical composition and thermal properties of maize starch from different varieties, showing that the starch characteristics can vary depending on genetic and environmental factors.

Mariscal Moreno et al. (10) examined the nutritional composition of maize tortillas made from different landraces and hybrids, highlighting the variability in protein, fiber, and mineral content between varieties. On the other hand, Rodríguez-Salinas et al. (4) and Salinas Moreno et al. (5) reported on the physicochemical properties and fatty acid composition of maize tortillas made from different colored varieties, finding that some genotypes had higher nutritional quality than others. Gaxiola-Cuevas (3), investigated the nutritional value and bioactive compounds of maize-based foods consumed by indigenous communities in Mexico, revealing the potential health benefits of these traditional foods. Finally, Urias-Lugo et al. (11, 12) examined the chemical composition, physical properties, and bioactive compounds of blue maize hybrids, showing that these varieties have a higher antioxidant capacity, phenolic content, and nutritional value than white or yellow maize.

Overall, these studies suggest considerable variability in the nutritional quality of maize genotypes and that the choice of the cultivar can impact the nutritional value of maize-based products such as tortillas. However, there is no integrated information about how the maize genotype influences the micro and macronutrients depending on if raw materials are landraces or high-yield hybrids. Therefore, this work will contribute to the existing literature by comparing the nutritional quality of tortillas made from landraces and high-yield hybrids and varieties, as well as dry masa flours, with a particular focus on micronutrients and bioactive compounds such as phenolics, minerals and antioxidants, but also on starch and protein fractions as one of the main macronutrients of tortilla and maize-based products. This information will give insights into the potential health benefits of using maize varieties in tortilla production that may be helpful for farmers, food processors and consumers.

## 2. Materials and methods

### 2.1. Maize genotypes

The maize genotypes (*Zea mays* L.) collected in a previous study were used (13). Among these samples were seven high producing hybrids and varieties (HPHV), five landraces, six hybrid maize mixtures that are commonly used in different regions of Mexico and six industrially produced and commercially available dry masa flours (DMF) from these blends (Table 1). The maize genotypes and flours were purchased from a national distributor based in Monterrey, Nuevo Leon in 2021.

**Table 1:** Maize Genotype employed in the preparation of tortillas.

Sample		Provenance	Crop Cycle/Year harvest	Color
H	Corteva P4279W	Campeche	Spring–Summer 2020	White
H	Corteva P4028W	Chiapas	Spring–Summer 2020	White
H	Bayer DEKALB 2037	Bajio	Spring–Summer 2020	White
H	Bayer Antilope/Berrendo	Jalisco	Spring–Summer 2020	White
H	Bayer DEKALB 4050	Sinaloa	Autumn-Winter 2020	White
V	INIFAP Quality Protein Maize	Bajio	Spring–Summer 2020	White
V	INIFAP High oil corn	Bajio	Spring–Summer 2020	White
L	Native Texhuaca	Estado de Mexico	Spring–Summer 2020	White
L	Native Blue	Estado de Mexico	Spring–Summer 2020	Blue
L	Olotillo	Oaxaca	Spring–Summer 2020	White
L	Serrano Mixe	Oaxaca	Spring–Summer 2020	Yellow
L	Chalqueño	Puebla	Spring–Summer 2020	White
M	Nuevo Leon	Sinaloa and Nuevo Leon	2020	White
M	Estado de Mexico	Bajio and Jalisco	2020	White
M	Bajio	Bajio and Sinaloa	2020	White
M	Jalisco	Jalisco	2019 and 2020	White
M	Veracruz	Sinaloa	2020	White
M	Chiapas	Sinaloa	2020	White
DMF	Nuevo Leon*	Sinaloa and Nuevo Leon	2020	White
DMF	Estado de Mexico	Bajio and Jalisco	2020	White
DMF	Bajio	Bajio and Sinaloa	2020	White
DMF	Jalisco*	Jalisco	2019 and 2020	White
DMF	Veracruz*	Sinaloa	2020	White
DMF	Chiapas	Sinaloa	2020	White

H, Hybrid maize; V, Maize varieties; L, Landraces; M, Hybrid mixtures; DMF, Dry masa flours. All DMF are enriched to comply with the government regulation NOM-247-SSA1-2008 [Secretaria de Economia (14)], with 4 mg of iron, 4 mg of zinc, 0.5 mg of Vitamin B1, 0.3 mg of Vitamin B2, 3.5 mg of Vitamin B3 and 0.2 mg of folic acid per 100 g of flour. *DMF with food preservatives.

### 2.2. Maize processing to nixtamal, masa, and tortilla

The same transformation of maize into nixtamal, masa and tortilla were followed as described by Acosta-Estrada et al. (13). Maize was lime-cooked with 1% lime and 3 parts of water at optimal cooking time (13) which is considered the sufficient time in which nixtamal reached 50% moisture after 15 h steeping. Masa was produced from nixtamal, which was ground in a commercial stone mill adjusted to yield fine masa, commonly used to produce tortillas. Masa was also produced from dry masa flour. The mixing times (1.17–1.90 min at 20 rpm) and the water absorptions (120–136%) were calculated with the help of a Mixolab (Mixolab 2, Chopin Technologies) (13, 15). To produce tortillas, the dough was continuously laminated and formed into circular pieces (12 cm of diameter and 21 g weight) with a commercial sheeter/former (Model V-25 comal en banda, Grupo Villamex, Mexico) and baked for 1 min at 148°C. Samples were frozen at-80°C and lyophilized (Virtis FM 25 EL 85, Gardiner, NY). Dry samples were ground with the Udy Mill (Cyclone Sample Mill no. 3010-014, Udy corporation, Fort Collins, CO; No. 80 US sieve) and stored until use.

### 2.3. Tortilla nutritional assessment

#### 2.3.1. Ferulic acid and antioxidant capacity

Extraction of free and bound phenolic compounds was performed using a slightly modified protocol from Acosta-Estrada et al. (16). One part of freeze-dried ground tortilla sample was mixed with 20 parts of ethanol (80%) for 10 min in a shaker (Incubator with orbital shaker, Mrc Laboratories, Israel) at 250 rpm and 25°C and then centrifuged (Thermofisher Scientific SL 16R, Waltham, MA) at 3,000 g (10 min and 4°C). The supernatant (yellow extract) was collected and identified as free phenolic extract and stored at −80°C until use. The resulting pellet was used to extract the bound phenolic compounds. Alkaline hydrolysis (10 parts NaOH 2 M) was performed for 1 h, the samples were then acidified with 2 M HCl to pH 2. The acidified samples were extracted with 50 parts of ethyl acetate and the fractions were evaporated to dryness (85 mbar, 25°C) (Genevac EZ-2 Series, SP Scientific, Warminster, PA, USA). The bound phenolics were resuspended in 50% methanol and samples stored at −80°C until use.

Phenolic extracts were analyzed through HPLC-PDA according to Acosta-Estrada et al. (16) method. Analyses of phenolic compounds were performed using an Agilent Series 1,260 Infinity HPLC system (Santa Clara, CA). Five μl of extracts were separated using a Zorbax SB-Aq reverse phase column, 3 mm × 150 mm. The elution gradient was performed with (A) 0.1% formic acid water and (B) acetonitrile at a flow rate of 0.6 mL/min. The gradient used was the following: 0/10 min, 15% B; 10/14 min, 58% of B; 14/20 min, 80% of B; 20/30 min, 100% B. Chromatograms were acquired at 320 nm and integrated using Open Lab CDS Chemstation LC A.02.13 software (Agilent Technologies). The identification and quantification of ferulic acid was based on the retention time of standard.

2,2-diphenyl-1-picrylhydrazyl (DPPH) radical scavenging activity was performed according to Karadag et al. (17). Briefly, 100 μL of phenolic tortilla extract was placed in a 96-well plate. Then, 100 μL of DPPH 100 μM (solubilized in 80% methanol) was added, and the solution was incubated for 30 min at 37°C in the absence of light. Finally, absorbance was measured at 517 nm using a microplate reader (Synergy HT, Bio-Tek, Winooski, VM) and 80% ethanol and 50% methanol were used as blank for free and bound extracts, respectively. Dilutions were prepared when needed. The antioxidant capacity was calculated using Eq. (1).


(1) $$DPPHScavenging\;\left(\%\right)=\frac{A_{Blank}-A_{Sample}}{A_{Blank}}x100$$

#### 2.3.2. Vitamins and minerals

Samples were characterized for ash according to AACC approved method 08-01.01 (2010). For mineral evaluation, 0.5 g of dry sample was digested with 10 mL of nitric acid 77% (v/v) in a microwave system (Mars 5 CEM, Matthews, NC, USA) for 10 min at 180°C. After digestion, the sample volume was adjusted to 25 mL with deionized water and filtered before performing the analysis. For this, an inductively coupled plasma mass spectrometry (ICP-MS) equipment (Agilent Technologies Model 7,800) was used under the following conditions: Purge time: 50s; stabilization time: 20s; Peak pattern: 3 points; Cycles: 100; Aftershocks: 3; Acquisition time: 19.3 s; Analytes: 23Na, 44Ca, 56Fe and 66Zn; Internal standard: 45Sc, 89Y.

The characterization and quantification of the B-complex vitamins was carried out according to Ramos-Parra et al. (18). Briefly, 1 g of sample was homogenized with 10 mL of 50 mM Na-Hepes/50 mM CHES, adjusted to pH 7.9 with HCl, 2% (w/v) Na-ascorbate, and 10 mM 2-mercaptoethanol. Homogenized samples were placed in a boiling water bath for 10 min. The resulting homogenates were centrifuged at 12,000 rpm for 10 min at 4°C, and the supernatants were collected. Throughout the preparation and analyses, all solutions were protected from exposure to light and stored at <5°C. For the chromatographic separation, an Atlantis T3 column with dimensions 4.6× 150 mm (5 μm, particle size) was used as the stationary phase, in a UPLC-H-class system (Waters ACQUITY) coupled to two detectors, photodiodes and fluorescence. Thiamine (B1) and niacin (B3) data were extracted at 270 nm; while for riboflavin (B2) and folic acid (B9), excitation lambdas of 423 and 295 nm, and emission lambdas at 525 and 358 nm, respectively, were used.

#### 2.3.3. Starch digestion

The total starch content of tortilla samples was determined according to the enzymatic protocol from Megazyme (Megazyme, Wicklow, Ireland) Total Starch Assay Kit (AA/AMG) K-TSTA-100 A (Megazyme, Wicklow, Ireland).

The *in vitro* starch digestibility of tortillas was determined according to the Englyst, Kingman, and Cummings (19) protocol with slight modifications (20). Tortilla samples (400 mg) were hydrated with 10 mL of deionized water and incubated in a shaking water bath for 20 min at 98°C and 200 rpm. Samples were cooled to 37°C and 8 mL of pepsin (5.21 mg/mL) added and incubated at 37°C and 200 rpm for 30 min. Afterwards, 8 mL of 0.5 M sodium acetate buffer (pH 5.2) were added and homogenized followed by 4 mL of an enzyme solution (pancreatin, amyloglucosidase, and invertase). At 20 and 120 min of reaction, 1 mL aliquots were mixed with 2 mL of 80% ethanol, and its glucose content quantified with the glucose oxidase-peroxidase reagent. The starch classification based on the rate of hydrolysis was: rapidly digestible starch (RDS) (digested within 20 min), slowly digestible starch (SDS) (digested between 20 and 120 min) and resistant starch (RS) (undigested after 120 min).

Samples were incubated in a shaking water bath for 30 min at 98°C and 300 rpm. Samples were cooled to 37°C followed by an enzymatic digestion. The percentage of hydrolyzed starch by Porcine pancreatic α-amylase at 30, 60, 90, 120, and 180 min was estimated. The hydrolysis index (HI) was calculated from the ratio between the area under the hydrolysis curve compared with a reference sample (white bread). The predicted GI (pGI) was estimated from the HI and values calculated using the Eq. (2) with a reported correlation coefficient of R = 0.89, *p* < 0.05.


(2) pGI=39.71+0.549(HI)

#### 2.3.4. *In vitro* protein digestibility and PDCAAS

Tortillas were analyzed for protein content according to approved AACCI method 46–13.01 (21) and for their complete amino acid profile following the Official HPLC Method 982.30 E (a, b, c) of the AOAC (22). The limiting essential amino acid was identified as the one with the lowest value in relation with the requirement for preschool children of the FAO/WHO/UNU expert consultation on protein and Amino Acid Requirements in Human Nutrition (23).

The *in vitro* protein digestibility of tortillas was evaluated according to the multienzyme pH-drop assay recommended by (24), The protocol has a correlation coefficient of R = 0.9 (*p* < 0.05). Briefly, the equivalent of 6.25 mg/L of protein was heated to 37°C and adjusted to a pH of 8.0, followed by the addition of a multi-enzyme solution containing trypsin (1.6 mg/mL), chymotrypsin (3.1 mg/mL), and protease from *S. griseus* (1.3 mg/mL). After the addition of the enzyme solution, the subsequent pH drop was recorded for 10 min. The *in vitro* protein digestibility was calculated as Eq. (3), where the ΔpH10 min is the change in pH in 10 min from the initial pH of about 8.0.


(3) $$\mathrm{IVDP}\%=210.46-18.1\Delta {\mathrm{pH}}_{10\;min}$$

Protein digestibility corrected amino acid score (PDCAAS), expressed as percentage units, were calculated by multiplying the chemical score of the limiting amino acid (lysine) and the *in vitro* protein digestibility.

### 2.4. Statistical analysis

Experiments were performed in triplicate and data was reported as mean ± standard deviation. Results were subjected to analysis of variance and differences among means were compared by Tukey tests at *p* < 0.05 in Minitab version 19.2020.1 (SAS Institute Inc. Cary, NC).

## 3. Results and discussion

### 3.1. Minor constituents: micronutrients and phytochemicals

Minor constituents such as vitamins and minerals (micronutrients) in maize play a vital role in basic physiology and nutrition, while phytochemicals exert positive bioactive effects on human health (25). There was no significant difference in the ferulic acid content of tortillas from evaluated groups (Table 2). Nonetheless, there were significant differences (*p* < 0.05) between the tortillas from individual genotypes in the concentration of ferulic acid. The genotype that yielded tortillas with the highest concentration of ferulic acid was hybrid Bayer DEKALB 2037 in both free (13.74 mg/100 g dw) and bound (84.40 mg/100 g dw), followed by tortillas from DMF Estado de Mexico (free 13.80 mg/100 g dw, bound 78.18 mg/100 g dw) and DMF Chiapas (free 11.19 mg/100 g dw, bound 54.63 mg/100 g dw) (Table 2). The tortilla samples with the lowest content of ferulic acid were from hybrid mixture Bajio (16.66 mg/100 g dw total ferulic acid) and DMF Jalisco (16.00 mg/100 g dw total ferulic acid) (Table 2). These findings are consistent with De la Parra et al. (26) who studied the phytochemical profile of tortillas made with maize of different colors with average total ferulic acid content of 75 mg/100 g dw. Ferulic acid is the major compound representing about 70% of the total phenolics (27).

**Table 2:** Ferulic acid content and antioxidant capacities determined by DPPH assay of tortillas from different maize genotypes.

Sample		Ferulic acid		Antioxidant Capacity	
		(mg/100 g) dw		% DPPH	
		Free	Bound	Free	Bound
H	Corteva P4279W	5.01 ± 0.01bcd	20.33 ± 3.61cd	55.92 ± 2.19a	125.00 ± 20.76a
H	Corteva P4028W	3.89 ± 1.42cd	19.01 ± 6.93cd	59.30 ± 1.79a	146.26 ± 6.44a
H	Bayer DEKALB 2037	13.74 ± 4.05a	84.40 ± 24.85a	62.39 ± 2.59a	161.95 ± 2.86a
H	Bayer Antilope/Berrendo	4.09 ± 1.15cd	23.19 ± 6.52cd	60.99 ± 8.96a	152.84 ± 31.49a
H	Bayer DEKALB 4050	3.17 ± 2.63d	16.67 ± 13.78d	58.03 ± 5.18a	141.70 ± 18.61a
V	INIFAP Quality Protein Maize	4.38 ± 0.52bcd	15.27 ± 1.80d	59.15 ± 9.56a	145.75 ± 34.35a
V	INIFAP High oil corn	8.78 ± 2.33abcd	42.86 ± 11.38bcd	61.55 ± 4.18a	154.35 ± 15.03a
L	Native Texhuaca	5.40 ± 3.16bcd	33.19 ± 19.39cd	65.21 ± 2.19a	167.51 ± 7.87a
L	Native Blue	2.41 ± 0.28d	18.92 ± 2.22cd	57.75 ± 5.18a	140.69 ± 18.61a
L	Olotillo	7.50 ± 1.08abcd	39.40 ± 5.68cd	63.24 ± 2.59a	165.49 ± 2.15a
L	Serrano Mixe	10.70 ± 3.36abc	34.46 ± 10.81cd	59.44 ± 3.59a	146.76 ± 12.88a
L	Chalqueño	7.65 ± 1.18abcd	43.36 ± 6.70bcd	59.58 ± 4.18a	147.27 ± 15.03a
M	Nuevo Leon	8.73 ± 1.88abcd	39.77 ± 8.55cd	55.92 ± 3.39a	134.11 ± 12.17a
M	Estado de Mexico	5.56 ± 1.97bcd	25.35 ± 8.97cd	56.48 ± 8.56a	136.14 ± 30.78a
M	Bajio	2.83 ± 0.26d	13.83 ± 1.25d	52.68 ± 5.18a	122.47 ± 18.61a
M	Jalisco	3.99 ± 0.62cd	20.94 ± 3.26cd	52.39 ± 9.16a	121.46 ± 32.92a
M	Veracruz	3.00 ± 0.34d	17.00 ± 1.95d	56.76 ± 1.79a	137.15 ± 6.44a
M	Chiapas	7.13 ± 2.31abcd	43.77 ± 14.18bcd	50.56 ± 7.77a	114.88 ± 27.91a
DMF	Nuevo Leon	2.44 ± 0.15d	14.96 ± 0.95d	52.11 ± 1.99a	120.45 ± 7.16a
DMF	Estado de Mexico	13.80 ± 0.69a	78.18 ± 3.90ab	59.58 ± 7.37a	147.27 ± 26.48a
DMF	Bajio	6.62 ± 0.14bcd	34.75 ± 0.73cd	61.27 ± 4.58a	153.34 ± 16.46a
DMF	Jalisco	2.72 ± 0.21d	13.28 ± 1.04d	56.90 ± 2.39a	137.65 ± 8.59a
DMF	Veracruz	6.02 ± 0.23bcd	27.43 ± 1.04cd	61.27 ± 7.77a	152.84 ± 28.63a
DMF	Chiapas	11.19 ± 0.92ab	54.63 ± 4.49abc	54.37 ± 3.19a	128.54 ± 11.45a
High producing hybrids and varieties		6.15 ± 3.81A	31.68 ± 25.03A	59.62 ± 2.23A	146.84 ± 11.73A
Landraces		6.73 ± 3.07A	33.87 ± 9.29A	61.04 ± 3.07A	153.54 ± 12.13A
Hybrids mixtures		5.21 ± 2.38A	26.78 ± 12.30A	54.13 ± 2.59B	127.70 ± 9.30B
Dry masa flours		7.13 ± 4.56A	37.21 ± 25.11A	57.58 ± 3.79AB	140.02 ± 13.53AB

H, Hybrid maize; V, Maize varieties; L, Landraces; M, Hybrid mixtures; DMF, Dry masa flours. Means with a different letter(s) within genotypes and groups of genotypes columns are statistically different (*p* < 0.05). dw, dry weight.

Moreover, results showed (Table 2) that bound ferulic acid corresponds to 83% of the total ferulic acid in the evaluated tortilla samples. Approximately 80% of the total maize phenolics are covalently linked to polysaccharides in hemicellulose in the cell walls of the pericarp and aleurone layers, crosslinking and strengthening maize cell walls providing defense mechanisms to both abiotic and biotic stress (28, 29). In maize, a positive correlation between maize oil quality and high contents of phenolic compounds under drought conditions has been demonstrated (30). Fat content in the same set of tortilla samples (13) had significant differences between tortillas from different genotypes, among which the ones with the highest oil content were High oil corn followed by Olotillo and DMF Bajio. At the time, differences in the concentration of ferulic acid in tortillas could be due to not only the genotype, but also epigenetic as stress signals are known to induce phenolic compounds to protect the plant system from oxidative stress (31).

Furthermore, phenolic compounds in maize have a wide range of therapeutic effects. They are antioxidant, anti-inflammatory, antiadipogenic, antidiabetic, and anticarcinogenic, among others (9, 32). Significant differences were observed in tortillas from evaluated groups in antioxidant activity measured by DPPH % inhibition. Although tortillas produced with high producing hybrids and varieties (HPHV) and landraces genotypes had the same antioxidant capacity (average 58.8 and 150.2% free and bound inhibition, respectively) (Table 2), tortillas from hybrid mixtures had lower antioxidant activity by 15% (54% free and 127% bound inhibition, respectively). Likewise, reports can be found in the literature where tortillas free phenolic compounds result in DPPH inhibition of between 34 to 45% (2, 33).

Interesting, when hybrid mixtures were processed into dry masa flours, resulting tortillas antioxidant capacity was again on par with tortillas from HPHV and landraces genotypes (197.60% total DPPH inhibition) (Table 2). Grain processing affects phenolic compounds and therefore their biological activity (25). Maize transformation to DMF involves thermal processing and milling, in which particle size is reduced, some phenolics accumulate in cellular vacuoles, and processing may release such unavailable phenolics (34). Similarly, thermal processing release bound phenolic acids by breaking down cell walls and browning during thermal processing increase antioxidant capacity by dissociating conjugated phenolic moiety followed by polymerization/oxidation reactions and the formation of other phenolics with higher therapeutic effect (16, 34).

**Figure 1:**
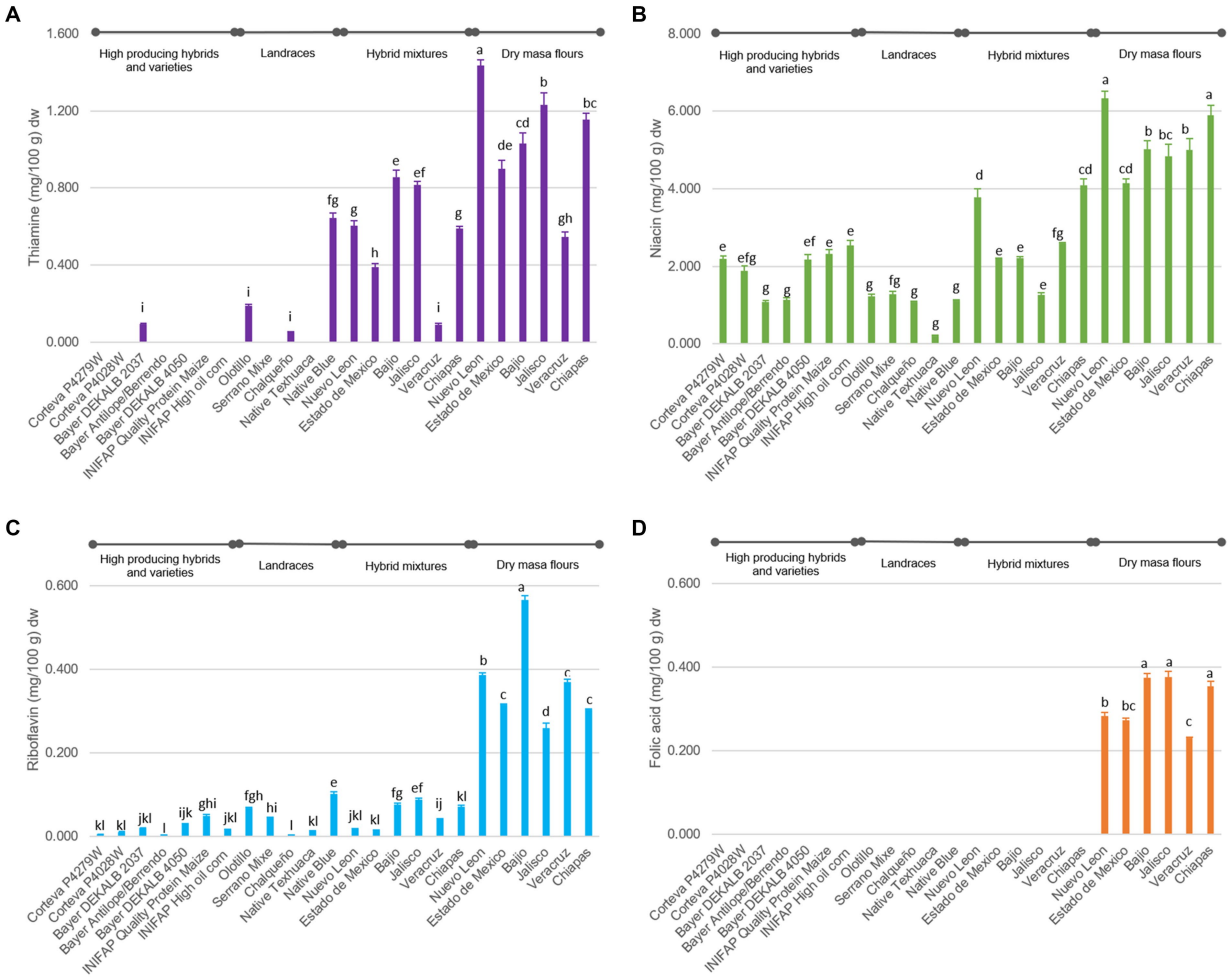
B complex vitamin contents in tortilla samples expressed in dry weight. **(A)** Thiamine. **(B)** Niacin. **(C)** Riboflavin. **(D)** Folic acid. The columns are the average of 3 repetitions and the bars represent the standard error of the mean. Different letters for the same type of vitamin are significantly different (Tukey, *p* < 0.05).

The overall vitamin contents of the tortillas were found in the range of 0.053–1.433, 0.003–0.566 and 0.216–6.330 mg/100 g dw for thiamine, riboflavin, and niacin, respectively (Figure 1), which are comparable to those reported in nutritional tables published by the USDA (35). All B-vitamins play important roles in maintaining good health. For example, deficiency in thiamine and niacin can lead to conditions called beriberi and pellagra, respectively (36). In terms of vitamin B1 (thiamine), it was observed that DMF tortilla samples had 11.2-fold and 3.5-fold more content than tortillas from HPHV and landraces, respectively (Figure 1A). Most tortillas from genotypes in the HPHV and landrace groups had undetectable thiamine values (Figure 1A). DMF tortilla samples had 2.7 and 5.3 times more niacin (B3) than HPHV and landraces tortillas, respectively. Between tortillas from landraces and HPHV, the latter contain 1.9 times more than the former (Figure 1B). Tortillas from DMF had 18.6 and 7.8 times more Riboflavin (vitamin B2) than from HPHV and landraces, respectively. Landrace tortillas contain 2.4 times more vitamin B2 than HPHV tortillas (Figure 1C).

Likewise, the tortillas samples elaborated with hybrid mixtures consistently contained higher levels of these vitamins compared to tortillas from landraces and HPHV (Figure 1). There are references in other crops where it has been reported that the landrace has more vitamins than the hybrid or improved crop. The possible explanation for this phenomenon is that, in crop improvement processes, the selection is usually based on agronomic characteristics or quality of the edible tissue. In the case of maize, a specific selection for micronutrients has been reported for increased vitamin A (in the form of carotenoids) in maize (37). The approach of manipulating a single gene, known as G-engineering and involving the introduction of GTPCHI, has been put into practice in various crops to increase folates including maize (38), and Mexican common bean (39). Also, a recent effort by Dong et al. (40) involved the modification of the thiamin biosynthesis pathway in rice in order to increase its thiamin content.

Large variations were detected in the concentration of individual vitamins from tortillas of different genotypes (Figure 1). The variation in the vitamin content can be associated with two sources: (a) Variation in the synthesis of vitamins in maize grains since it depends on and is affected by the genotype-environment interaction (41). Environmental variables such as temperature, precipitation, soil pH and texture, organic matter, among others, influence the concentration of nutrients in crops (42, 43). (b) Different and partial degradation of vitamins in postharvest handling: storage time, humidity, temperature, processing, etc. (44).

On the other hand, folic acid was not detected in any of the samples other than in tortillas produced by DMF (Figure 1D). The dry masa flours are enriched to comply with the government regulation NOM-247-SSA1-2008 (14), with 0.5 mg of Vitamin B1, 0.3 mg of Vitamin B2, 3.5 mg of Vitamin B3 and 0.2 mg of folic acid per 100 g of flour. Fat-soluble and water-soluble vitamins are mainly found in maize germ and aleurone, respectively (25); the removal of these parts during decortication, degermed, etc… significantly reduces the amount of vitamins (45), therefore, the fortification of cereal-based foods around the world is aimed at restoring certain vitamins that are essential for human nutrition (46). Most fortification programs restore and/or increase the amounts of thiamine, riboflavin, niacin, and folic acid (25). The vitamin contents determined in the DMF tortilla samples correspond to the amount of added vitamin in dry masa flour plus the amount of vitamin from maize on average.

Previous publications with the same set of samples found that tortillas produced from DMF contained 22.6% more ash than the other tested groups (13). When assessing each individual mineral, it was found that the DMF tortilla samples contained 1.75 times more sodium (Na) and 2.75 times more iron (Fe) than the other groups of samples (Table 3). Maize products can contain higher iron levels due to contamination from processing equipment (47), among others. Dunn et al. (48) previously established iron values for commercial and homemade maize tortillas of 2.68 and 0.48 mg/100 g dw, respectively, similar to our tortillas made from HPHV and landraces genotypes.

**Table 3:** Mineral content of maize tortillas as expressed in mg/100 g of dry weight.

Sample		Na	Ca	Fe	Zn
H	Corteva P4279W	7.76 ± 0.35defgh	202.42 ± 1.88a	2.79 ± 0.056ghij	2.69 ± 0.10efgh
H	Corteva P4028W	9.20 ± 0.61cde	186.28 ± 0.48bc	2.79 ± 0.103ghij	2.38 ± 0.02efgh
H	Bayer DEKALB 2037	6.26 ± 0.07hijk	181.28 ± 1.48cd	2.19 ± 0.072j	2.50 ± 0.03efgh
H	Bayer Antilope/Berrendo	8.39 ± 0.27def	179.16 ± 1.38d	2.58 ± 0.072hij	3.01 ± 0.15cdefgh
H	Bayer DEKALB 4050	7.33 ± 0.24efghij	136.45 ± 1.84jk	2.13 ± 0.048j	2.76 ± 0.08defgh
V	INIFAP Quality Protein Maize	6.04 ± 0.27hijk	147.66 ± 1.97hi	7.00 ± 0.006cd	3.07 ± 0.15cdefgh
V	INIFAP High oil corn	6.39 ± 0.32ghijk	172.65 ± 0.31e	3.30 ± 0.114fghi	2.66 ± 0.21efgh
L	Native Texhuaca	7.61 ± 0.16defghi	183.40 ± 1.76bcd	4.90 ± 0.201e	4.13 ± 0.05cd
L	Native Blue	5.53 ± 0.14jk	125.46 ± 0.72l	3.47 ± 0.134fgh	3.68 ± 0.01cde
L	Olotillo	7.20 ± 0.07fghijk	148.98 ± 1.61h	3.65 ± 0.175fg	3.23 ± 0.13cdefg
L	Serrano Mixe	6.57 ± 0.37fghijk	204.66 ± 0.17a	2.26 ± 0.052j	3.39 ± 0.13cdef
L	Chalqueño	8.33 ± 0.01defg	156.49 ± 1.21g	2.98 ± 0.036fghij	4.22 ± 0.08c
M	Nuevo Leon	6.12 ± 0.28hijk	158.59 ± 0.43fg	3.91 ± 0.138f	2.96 ± 0.24cdefgh
M	Estado de Mexico	5.72 ± 0.13ijk	171.48 ± 0.29e	2.96 ± 0.133fghij	2.65 ± 0.04efgh
M	Bajio	6.24 ± 0.41hijk	135.37 ± 0.01k	3.39 ± 0.042fghi	1.91 ± 0.06gh
M	Jalisco	BLQ	142.06 ± 0.38ij	3.090 ± 050fghij	1.810 ± 0.03h
M	Veracruz	7.83 ± 0.38defgh	138.09 ± 0.14jk	2.47 ± 0.002ij	2.25 ± 0.08fgh
M	Chiapas	5.34 ± 0.08k	187.49 ± 1.08b	2.52 ± 0.044hij	2.41 ± 0.10efgh
DMF	Nuevo Leon	16.21 ± 0.36a	102.00 ± 1.56n	7.78 ± 0.373c	7.56 ± 0.40a
DMF	Estado de Mexico	10.48 ± 0.68c	163.35 ± 0.64f	10.80 ± 0.480a	7.60 ± 0.63a
DMF	Bajio	9.36 ± 0.02cd	136.38 ± 0.30jk	9.57 ± 0.033b	7.98 ± 0.45a
DMF	Jalisco	15.44 ± 0.32a	98.98 ± 0.48n	7.83 ± 0.241c	6.03 ± 0.36b
DMF	Veracruz	7.69 ± 0.50defgh	159.47 ± 0.29fg	6.74 ± 0.069d	8.01 ± 0.33a
DMF	Chiapas	12.94 ± 0.57b	111.56 ± 0.26m	10.84 ± 0.262a	8.00 ± 0.40a
High producing hybrids and varieties		7.34 ± 1.19B	172.27 ± 22.81A	3.25 ± 1.70B	2.73 ± 0.25C
Landraces		7.05 ± 1.06B	163.80 ± 30.81AB	3.45 ± 0.97B	3.73 ± 0.44B
Hybrids mixtures		6.25 ± 0.95B	155.51 ± 20.87AB	3.06 ± 0.54B	2.33 ± 0.44C
Dry masa flours		12.02 ± 3.41A	128.62 ± 28.62B	8.93 ± 1.73A	7.53 ± 0.76A

H, Hybrid maize; V, Maize varieties; L, Landraces; M, Hybrid mixtures; DMF, Dry masa flours. Means with a different letter(s) within genotypes and groups of genotypes columns are statistically different (*p* < 0.05). BLQ, Below the limit of quantification.

Zinc (Zn) in the evaluated tortillas was present in higher concentration in DMF tortillas followed by landraces tortillas and those with the lowest amount were tortillas from HPVP and hybrid mixtures with values of 7.53, 3.73, and 2.53 mg/100 g, respectively (Table 3). Commercial tortillas in literature (48) had an average of 2.5 mg Zn/100 g dw on par with our results for most tortillas from genotypes tested. The concentration of zinc in maize can be affected by phenotypic factors such as soil fertility (49) and processing that enhance its bioaccessibility and bioavailability by removing inhibitors (e.g., phytates) or releasing nutrients from the food matrix (50).

Iron and zinc are the most common micronutrient deficiencies in cereal-based diets. This is the main reason why cereal fortification programs around the world supplement these essential minerals (51). In Mexico, the dry masa flours are enriched, to comply with the government regulation NOM-247-SSA1-2008 (14), with 4 mg of iron and 4 mg of zinc for every 100 g of flour. Even if we subtract the addition of iron and zinc, DMF tortilla samples are still among the top 5 samples with the highest iron content with a range of 5.57–6.84 mg/100 g dw (namely DMF Chiapas, DMF Estado de Mexico and DMF Bajio), only surpassed by tortillas from the variety Quality protein maize (7 mg/100 g) (Table 3). Similarly, DMF tortilla samples are still among the top 5 samples with the highest zinc with an average content of 3.99 mg/100 g (namely DMF Chiapas, DMF Veracruz and DMF Bajio) (Table 3). The pericarp, germ and aleurone layer are the anatomical parts that contain the highest concentration of most minerals (45). In a previously published article (13), the samples with the highest pericarp retention after nixtamalization were: quality protein maize followed by a mixture of hybrids with which DMF Bajio and DMF Veracruz were made.

On the other hand, DMF tortilla samples on average contained 0.75 times less calcium than the HPHV tortilla samples. Differences were observed in the concentration of calcium in the tortillas from individual genotypes. Tortillas from Serrano Mixe landrace (204.66 mg/100 g) and the hybrid Corteva P4279W (202.42 mg/100 g) were the samples with the highest calcium concentrations. Results confirm an almost identical value of calcium concentration for tortillas from high quality corn protein, obtaining 147 mg/100 g dw (Table 3) versus 144 mg/100 g in literature (52) with same concentration/procedure.

### 3.2. Macronutrientes: starch and protein

Just as starch and its interaction with other molecules (fats, proteins, ferulic acid) influence the quality of tortilla (13), the type of starch influences the nutritional profile of the tortilla. The rapidly digestible starch is hydrolyzed and absorbed within the first 20 min after ingestion, which rapidly increases glucose levels in the bloodstream. In comparison, slowly digestible starch causes the glucose produced to be gradually absorbed into the blood, so there are no abrupt increases in glucose concentration that generate a high demand for insulin (53). The rapidly digestible and slowly digestible starch fractions were found in a range of 65.52 to 84.35% and 12.94–31.26%, respectively (Table 4) and no significant differences were found between the studied groups but there was significant difference between individual samples.

**Table 4:** Starch type analysis in maize tortillas and predicted glycemic index.

Sample		Rapidly digested starch (%)	Slowly digestible starch (%)	Resistant starch* (%)	Predicted glycemic index*
H	Corteva P4279W	74.59 ± 0.86hij	22.59 ± 0.13def	2.81 ± 0.91	90.25 ± 0.50
H	Corteva P4028W	84.35 ± 0.31a	12.94 ± 0.16l	2.71 ± 0.86	92.09 ± 0.77
H	Bayer DEKALB 2037	81.94 ± 0.93abcd	14.95 ± 0.84kl	3.11 ± 0.13	91.56 ± 0.43
H	Bayer Antilope/Berrendo	76.98 ± 0.09ghi	20.70 ± 0.03efgh	2.32 ± 0.22	90.79 ± 0.56
H	Bayer DEKALB 4050	77.79 ± 0.88efgh	18.71 ± 0.80ghij	3.50 ± 0.20	90.72 ± 0.67
V	INIFAP Quality Protein Maize	79.46 ± 0.37cdefg	17.13 ± 0.52ijk	3.41 ± 0.51	91.05 ± 0.85
V	INIFAP High oil corn	74.53 ± 0.94hij	21.77 ± 0.09efg	3.69 ± 0.63	90.08 ± 0.79
L	Native Texhuaca	74.24 ± 0.40ij	23.70 ± 0.85de	2.06 ± 0.01	90.33 ± 0.73
L	Native Blue	66.77 ± 0.85lm	30.25 ± 0.28ab	2.98 ± 0.86	88.77 ± 0.74
L	Olotillo	71.34 ± 0.01jk	25.70 ± 0.74cd	2.95 ± 0.90	89.62 ± 0.00
L	Serrano Mixe	76.31 ± 0.35ghi	21.37 ± 0.95efgh	2.32 ± 0.20	90.66 ± 0.69
L	Chalqueño	82.51 ± 0.23abc	15.16 ± 0.13kl	2.33 ± 0.09	91.81 ± 0.16
M	Nuevo Leon	80.76 ± 0.44bcdef	17.12 ± 0.87ijk	2.12 ± 0.62	91.53 ± 0.16
M	Estado de Mexico	80.89 ± 0.99bcde	15.80 ± 0.01jkl	3.31 ± 0.32	91.33 ± 0.24
M	Bajio	66.42 ± 0.30m	31.26 ± 0.98a	2.31 ± 0.04	88.82 ± 0.15
M	Jalisco	82.91 ± 0.43ab	13.42 ± 0.16l	3.67 ± 0.70	91.64 ± 0.08
M	Veracruz	65.52 ± 0.22m	30.52 ± 0.94ab	3.96 ± 0.22	88.35 ± 0.17
M	Chiapas	69.78 ± 0.61kl	27.37 ± 0.91bc	2.85 ± 0.40	89.35 ± 0.79
DMF	Nuevo Leon	79.02 ± 0.92defg	18.07 ± 0.86hijk	2.92 ± 0.27	91.05 ± 0.23
DMF	Estado de Mexico	77.47 ± 0.11fghi	18.75 ± 0.21ghij	3.78 ± 0.37	90.61 ± 0.37
DMF	Bajio	77.25 ± 0.43ghi	19.73 ± 0.78fghi	3.02 ± 0.74	90.71 ± 0.70
DMF	Jalisco	83.12 ± 0.10ab	13.57 ± 0.15l	3.30 ± 0.86	91.75 ± 0.40
DMF	Veracruz	81.71 ± 0.76abcd	14.69 ± 0.26kl	3.60 ± 0.04	91.43 ± 3.60
DMF	Chiapas	80.94 ± 0.04bcde	16.39 ± 0.31ijkl	2.68 ± 0.04	91.46 ± 0.71
High producing hybrids and varieties		78.52 ± 3.67A	18.40 ± 3.60A	3.08 ± 0.49	90.93 ± 0.71
Landraces		74.23 ± 5.85A	23.24 ± 10.72A	2.53 ± 0.41	90.24 ± 36.85
Hybrids mixtures		74.38 ± 7.99A	22.58 ± 8.01A	3.04 ± 0.74	90.17 ± 1.49
Dry masa flours		79.92 ± 2.38A	16.87 ± 2.41A	3.22 ± 0.42	91.17 ± 0.45

H, Hybrid maize; V, Maize varieties; L, Landraces; M, Hybrid mixtures; DMF, Dry masa flours. Means with a different letter(s) within genotypes and groups of genotypes columns are statistically different (*p* < 0.05). *Sample without significant difference between samples or between groups.

Tortillas from hybrid mixtures Bajio and Veracruz, and landrace native Blue showed the highest amounts of slowly digestible starch (Table 4) whereas from hybrid Corteva P4028W, hybrid mixture Jalisco and DMF Jalisco contained the highest rapidly digestible starch contents (Table 4). Vice versa, tortillas from hybrid mixture Bajio and Veracruz, and landrace native Blue showed the lowest amount of rapidly digestible starch (Table 4) whereas from hybrid Corteva P4028W, hybrid mixture Jalisco and DMF Jalisco contained the lowest slowly digestible starch content (Table 4). The tortilla samples showed starch digestion fractions similar to previous reports (54) with the typical high amounts of rapidly digestible starch (>52%), relatively low slowly digestible starch (<18.5%).

Resistant starch is a type of starch that is not completely digested in the small intestine of our body, so when reaches the hind gut it acts in a similar way to prebiotic fiber. Variability was found between tortilla samples (2.06–3.96%) with an average value of 3% across samples (Table 4). In the same sense, previous research has shown that resistant starch is present in traditional tortillas and commercial tortillas at 5.79 and 11.36%, respectively (54).

The glycemic index ranks carbohydrate-containing foods according to their ability to raise blood glucose levels. When evaluating the *in vitro* glycemic index (Table 4), all tortillas samples showed predicted glycemic indexes (pGI) of 88.35 up to 92.09, which classify them as high glycemic index foods. Studies have shown that pGI values of white and blue tortillas were 91 and 85.5, respectively (55). Although it has a high glycemic index value, it has already been shown that digestive enzymes do not hydrolyze all the starch present in tortillas and that the starch digestion rate is more important to determine health benefits (54).

**Figure 2:**
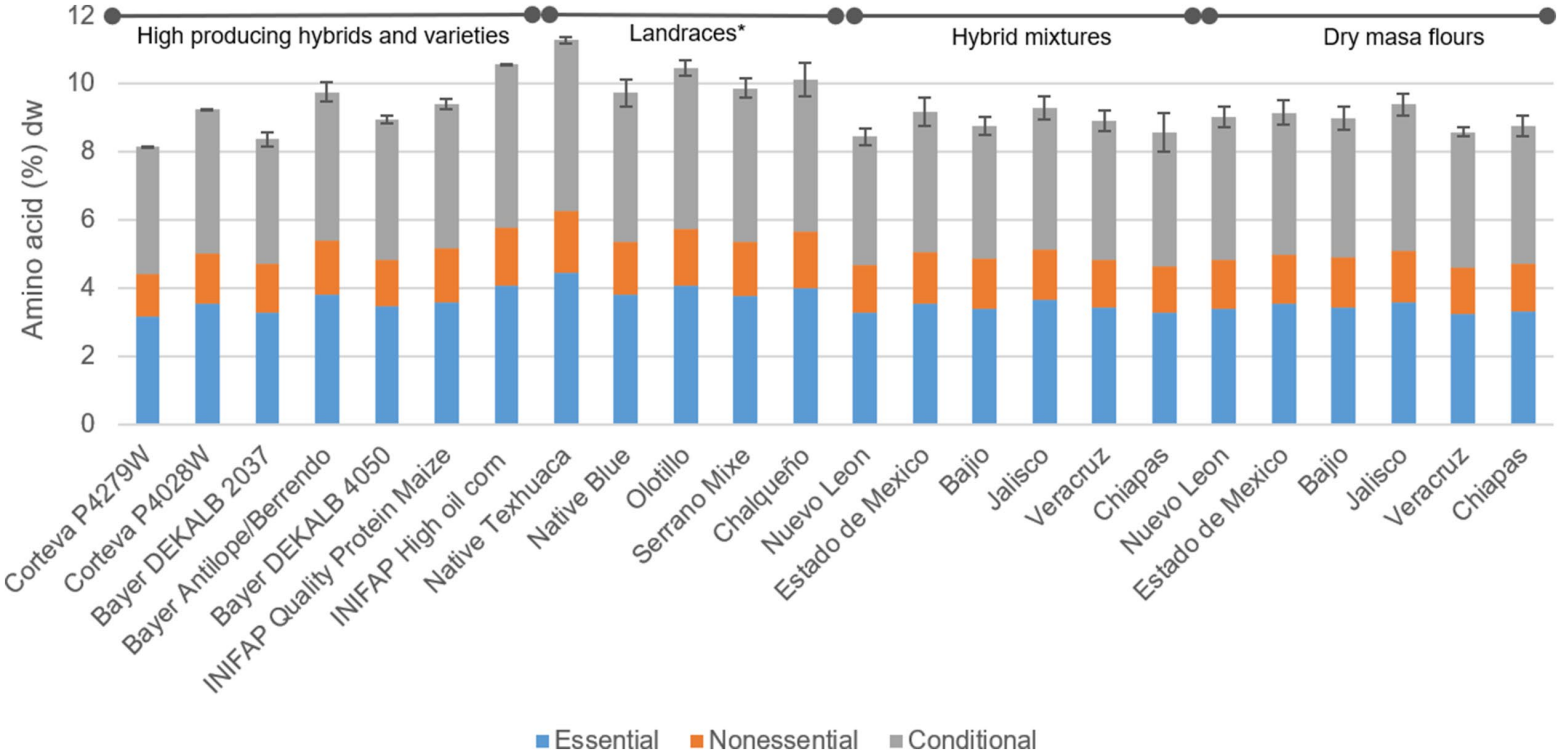
Distribution of amino acids in maize tortillas (dry basis). Means with asterisk (*) within groups of genotypes are statistically different (*p* < 0.05).

The tortilla samples produced with landraces on average contained 14% higher protein versus the average tortillas from HPHV, DMF and Hybrid Mixes (Figure 2). When evaluating tortillas from the individual genotypes, protein ranged from 8.15 to 11.28%, the highest associated to the Native Texhuaca landrace (11.28 ± 0.10%) and the lowest Corteva P4279W (8.15 ± 0.02%) (Figure 2). Maize proteins are deficient in some essential amino acids, such as tryptophan (0.07% dry base) and lysine (Supplementary Table S1), which reduces the quality and its nutritional properties (56). The limiting amino acid in maize tortillas is lysine, which was found at average values of 0.28 g/100 g dry weight (Supplementary Table S1). Being the tortilla a fundamental food in the Mexican diet, this characteristic of maize worsens the malnutrition problem in communities that base their diet on tortillas and related products. There are efforts to fortify tortillas with tryptophan and lysine and combat it in developing countries, where protein-deficient diets are common, and essential amino acid deficiency can contribute to a condition called kwashiorkor (57).

No significant differences were found in the lysine content in the evaluated groups, but there was significant difference between individual samples. Unsurprisingly, tortillas with the highest amount of lysine were from high-quality protein with 75.16 g/100 g protein, followed by the mixture and DMF Chiapas (Table 5). When comparing tortillas from these two genotypes with the one with the lowest lysine content (landrace Olotillo) it was found that these first two contained 39.18 and 23.7% more lysine, respectively. The evaluated tortillas contained similar amounts of lysine previously reported (52). Differences in the amino acid composition in grains had been attributed to growing environmental conditions (e.g., CO_2_ concentration and temperature), genetic makeup, and the use of fertilizers (58).

**Table 5:** Limiting amino acid content, *in vitro* protein digestibility and PDCAAS in tortillas made with different maize genotypes.

Sample		Lysine (g/100 g protein)*	*In vitro* protein digestibility (%)	PDCAAS
H	Corteva P4279W	63.45 ± 0.181cdef	61.41 ± 1.66ijk	38.97 ± 0.84jk
H	Corteva P4028W	58.19 ± 0.108ijk	62.66 ± 0.41hijk	36.46 ± 0.80kl
H	Bayer DEKALB 2037	62.42 ± 0.703defg	68.46 ± 1.24fghij	42.74 ± 0.19ghij
H	Bayer Antilope/Berrendo	57.3 ± 0.671jkl	59.34 ± 1.24jk	34.00 ± 0.82lm
H	Bayer DEKALB 4050	64.33 ± 0.241bcde	73.03 ± 0.83cdefgh	46.98 ± 0.71def
V	INIFAP Quality Protein Maize	75.16 ± 0.122a	70.95 ± 4.56efghi	53.33 ± 1.12c
V	INIFAP High oil corn	56.59 ± 0.826kl	69.29 ± 0.41efghij	39.21 ± 1.18ijk
L	Native Texhuaca	58.26 ± 0.383ijk	79.25 ± 0.41bcde	46.17 ± 0.93efg
L	Native Blue	62.92 ± 0.974cdef	96.27 ± 2.49a	60.57 ± 0.43a
L	Olotillo	54 ± 0.611l	72.20 ± 0.83defgh	38.99 ± 0.26jk
L	Serrano Mixe	58.47 ± 0.783hijk	77.59 ± 0.41bcdef	45.37 ± 0.88efg
L	Chalqueño	59.16 ± 0.002ghijk	46.89 ± 0.41l	27.74 ± 0.85n
M	Nuevo Leon	59.9 ± 0.219fghijk	52.28 ± 0.83kl	31.31 ± 0.63mn
M	Estado de Mexico	62.55 ± 0.462cdefg	68.88 ± 0.00efghij	43.08 ± 0.69fghi
M	Bajio	62.75 ± 0.852cdefg	65.56 ± 0.83ghij	41.14 ± 1.03hij
M	Jalisco	62.12 ± 0.793defgh	68.05 ± 1.66fghij	42.27 ± 1.08ghij
M	Veracruz	65.26 ± 0.900bcd	68.05 ± 0.00fghij	44.41 ± 0.28efgh
M	Chiapas	66.14 ± 0.837bc	65.98 ± 1.24ghij	43.63 ± 0.28fgh
DMF	Nuevo Leon	64.72 ± 0.952bcd	82.99 ± 0.83bc	53.71 ± 0.77bc
DMF	Estado de Mexico	63.28 ± 0.043cdef	83.82 ± 0.00b	53.04 ± 0.14c
DMF	Bajio	61.71 ± 0.977defghi	82.16 ± 4.98bcd	50.7 ± 0.12cd
DMF	Jalisco	60.86 ± 0.760efghij	94.61 ± 2.49a	57.58 ± 0.06ab
DMF	Veracruz	63.91 ± 0.431bcde	74.69 ± 1.66bcdefg	47.73 ± 0.55de
DMF	Chiapas	67.45 ± 0.529b	74.69 ± 1.66bcdefg	50.37 ± 0.15cd
High producing hybrids and varieties		62.49 ± 6.39A	66.45 ± 5.26B	41.67 ± 6.64B
Landraces		58.56 ± 3.17A	74.44 ± 17.84AB	43.77 ± 11.94AB
Hybrids mixtures		63.12 ± 2.26A	64.80 ± 6.27B	40.97 ± 4.87B
Dry masa flours		63.66 ± 2.34A	82.16 ± 7.35A	52.19 ± 3.39A

H, Hybrid maize; V, Maize varieties; L, Landraces; M, Hybrid mixtures; DMF, Dry masa flours. Means with a different letter(s) within genotypes and groups of genotypes columns are statistically different (*p* < 0.05). *Limiting amino acid percentage required. PDCAAS: Protein digestibility-corrected amino acid score. Reference pattern: Preschool children.

The highest protein quality evaluated by protein digestibility corrected amino acid score (PDCAAS) was found in the tortillas produced from DMF, with an average value of 52.19 (Table 5) that represents 27, 25 and 19% more than tortillas produce by hybrids mixture, HPHV and landraces, respectively; on the other hand, tortillas from landraces had the greatest variability with values between 27.74 (landrace Chalqueño) and 60.57 (landrace ative Blue) (Table 5), having the tortilla samples with the highest and lowest PDCAAS. These values agree with those reported in the literature for maize tortillas with a PDCAAS of 35.53 (57). A food with PDCAAS values below 50 is considered poor according to the FAO; 79.8% of the evaluated tortillas were considered to have poor quality protein and 29.2% to have moderate quality protein.

The PDCAAS of the tortillas was associated to its *in vitro* protein digestibility (IVPD). When evaluating the IVPD of tortilla groups, it was found that the DMF tortilla samples had a digestibility of 82.16% compared to 74.44% from landraces and 65% for HPHV and hybrid mixtures (Table 5). The IVPD of maize tortillas in the literature is 72.5% (57). The IVPD specifies the protein quantity absorbed by an organism relative to the consumed amount and depends on the protein structure, previous processing (e.g., cooking, drying, milling) and the presence of antinutritional factors (e.g., phytates present in maize) (59). Phytates are reduced by up to 21% during nixtamalization or lime-cooking (48) improving IVPD and therefore PDCAAS of tortillas.

## 4. Conclusion

Among the nutritional characteristics analyzed no significant differences were found in ferulic acid, starch (rapidly digestible and slowly digestible) and lysine contents between the evaluated groups. Tortillas produced from dry masa flour presented the highest protein quality (PDCAAS), had the highest vitamins (B1, B2, B23), sodium, iron, and zinc contents, and were the only samples with folic acid. Hybrids and varieties had the highest antioxidant capacity and calcium contents. Landraces had the highest protein contents and antioxidant capacity. Although some nutritional aspects are associated with the maize genotype (e.g., amino acid composition), most of the nutritional aspects evaluated (vitamins, minerals, ferulic acid) can be associated with factors external to the genetic makeup and others can be modified during processing (Fe, Zn, *in vitro* protein digestibility, PDCAAS). There is no maize genotype that is more nutritious than another, since some may be high in protein but low in digestibility and PDCAAS (e.g., landraces) or may be high in phytochemical compounds but low in antioxidant activity (e.g., Hybrids). There are genotypes that can be combined as mixtures to design superior nutritional tortillas and related products for populations that highly consume them and improve their human health. It is important to note that there are limitations to this study as it did not consider the agronomic management and post-harvest handling practices of the different corn genotypes previously their acquisition. Further investigations could evaluate the effect of nixtamalization process variables on protein digestibility and bioavailability and their optimization to produce dry masa flours.

## Data availability statement

The raw data supporting the conclusions of this article will be made available by the authors, without undue reservation.

## Author contributions

BA-E, SS-S, and CC-H contributed to conception and design of the study. BA-E and CC-H organized the database and performed the statistical analysis. BA-E wrote the first draft of the manuscript. CC-H wrote sections of the manuscript. SS-S revised it critically for important intellectual content. All authors contributed to the article and approved the submitted version.

## Conflict of interest

The authors declare that the research was conducted in the absence of any commercial or financial relationships that could be construed as a potential conflict of interest.

## Publisher’s note

All claims expressed in this article are solely those of the authors and do not necessarily represent those of their affiliated organizations, or those of the publisher, the editors and the reviewers. Any product that may be evaluated in this article, or claim that may be made by its manufacturer, is not guaranteed or endorsed by the publisher.
