# Supplementary material for: Nutritional assessment of nixtamalized maize tortillas produced from dry masa flour, landraces, and high yield hybrids and varieties
Source: Front Nutr. 2023 Jul 6;10:1183935. doi: 10.3389/fnut.2023.1183935 (PMC10358733; doi:10.3389/fnut.2023.1183935)
Supplement: Supplementary file 1 [file Table_1.pdf]

Table S1. Aminogram of tortillas produced with different maize genotypes.

Amino acids (g/100 g dw)	Corteva P4279W	Corteva P4028W	Bayer DEKALB 2037	Bayer Antilope / Berrendo	Bayer DEKALB 4050	INIFAP Quality Protein Maize	INIFAP High oil corn	Olotillo	Serrano Mixe	Chalqueño	Native Texhuaca	Native Blue
	H	H	H	H	H	V	V	L	L	L	L	L
Taurine	0.14	0.20	0.18	0.17	0.14	0.18	0.19	0.19	0.16	0.17	0.17	0.14
Hydroxyproline	0.02	0.02	0.03	0.02	0.02	0.03	0.02	0.02	0.03	0.02	0.01	0.01
Aspartic Acid	0.50	0.55	0.54	0.63	0.55	0.70	0.66	0.64	0.60	0.65	0.72	0.64
Threonine	0.29	0.32	0.29	0.33	0.32	0.35	0.36	0.37	0.35	0.35	0.39	0.34
Serine	0.36	0.41	0.38	0.44	0.40	0.41	0.48	0.47	0.45	0.46	0.51	0.46
Glutamic Acid	1.54	1.79	1.64	1.95	1.72	1.72	2.09	2.07	1.91	2.04	2.25	1.90
Proline	0.74	0.85	0.73	0.86	0.83	0.87	0.97	0.96	0.89	0.90	1.02	0.86
Lanthionine	0.02	0.00	0.02	0.01	0.01	0.01	0.02	0.01	0.02	0.01	0.02	0.00
Glycine	0.32	0.35	0.30	0.33	0.34	0.39	0.37	0.35	0.37	0.35	0.39	0.36
Alanine	0.60	0.69	0.65	0.75	0.66	0.65	0.80	0.79	0.74	0.79	0.88	0.75
Cysteine	0.19	0.21	0.16	0.18	0.18	0.22	0.21	0.23	0.23	0.22	0.23	0.20
Valine	0.42	0.46	0.42	0.48	0.46	0.51	0.53	0.51	0.50	0.50	0.57	0.49
Methionine	0.20	0.21	0.15	0.16	0.16	0.14	0.17	0.28	0.19	0.24	0.25	0.22
Isoleucine	0.30	0.34	0.32	0.38	0.33	0.35	0.40	0.39	0.36	0.39	0.45	0.37
Leucine	0.98	1.16	1.10	1.32	1.11	1.05	1.40	1.37	1.23	1.36	1.51	1.26
Tyrosine	0.19	0.22	0.14	0.22	0.22	0.17	0.23	0.25	0.25	0.15	0.20	0.21
Phenylalanine	0.38	0.44	0.44	0.52	0.45	0.45	0.55	0.52	0.48	0.52	0.59	0.50
Hydroxylysine	0.00	0.00	0.01	0.01	0.01	0.01	0.01	0.01	0.01	0.01	0.01	0.01
Ornithine	0.01	0.01	0.01	0.01	0.01	0.01	0.01	0.01	0.01	0.01	0.01	0.01
Lysine*	0.25	0.26	0.25	0.27	0.28	0.34	0.29	0.27	0.28	0.29	0.32	0.29
Histidine	0.27	0.30	0.24	0.28	0.29	0.34	0.32	0.29	0.32	0.28	0.30	0.26
Arginine	0.36	0.39	0.31	0.38	0.40	0.45	0.42	0.39	0.41	0.34	0.40	0.38
Tryptophan	0.06	0.07	0.06	0.06	0.06	0.06	0.07	0.08	0.08	0.08	0.08	0.07
Total	8.15	9.25	8.36	9.78	8.95	9.4	10.59	10.46	9.87	10.14	11.28	9.69

H = Hybrid maize; V= Maize varieties; L = Landraces; M= Hybrid mixtures; DMF = Dry masa flours. *Lysine is the limiting amino acid in the evaluated corn tortillas.

Table S1. Continuation

Amino acids (g/100 g dw)	Nuevo León	Estado de México	Bajío	Jalisco	Veracruz	Chiapas	Nuevo León	Estado de México	Bajío	Jalisco	Veracruz	Chiapas
	M	M	M	M	M	M	DMF	DMF	DMF	DMF	DMF	DMF
Taurine	0.18	0.18	0.18	0.14	0.15	0.13	0.14	0.14	0.15	0.13	0.14	0.15
Hydroxyproline	0.01	0.01	0.02	0.03	0.03	0.02	0.02	0.03	0.03	0.02	0.03	0.02
Aspartic Acid	0.55	0.59	0.57	0.61	0.56	0.56	0.59	0.60	0.60	0.62	0.56	0.57
Threonine	0.30	0.33	0.31	0.32	0.31	0.30	0.31	0.32	0.32	0.33	0.31	0.31
Serine	0.39	0.42	0.39	0.43	0.41	0.40	0.41	0.42	0.40	0.43	0.4	0.4
Glutamic Acid	1.65	1.77	1.70	1.83	1.70	1.63	1.73	1.78	1.72	1.83	1.63	1.64
Proline	0.73	0.79	0.77	0.83	0.77	0.72	0.80	0.79	0.81	0.85	0.78	0.79
Lanthionine	0.02	0.01	0.01	0.00	0.00	0.01	0.00	0.00	0.00	0.01	0	0.01
Glycine	0.31	0.33	0.33	0.33	0.34	0.34	0.35	0.34	0.33	0.33	0.34	0.35
Alanine	0.64	0.69	0.67	0.71	0.65	0.64	0.67	0.68	0.66	0.71	0.62	0.63
Cysteine	0.17	0.19	0.19	0.19	0.18	0.18	0.21	0.19	0.19	0.19	0.18	0.2
Valine	0.41	0.44	0.44	0.47	0.45	0.42	0.43	0.45	0.44	0.45	0.42	0.43
Methionine	0.17	0.18	0.17	0.16	0.17	0.18	0.18	0.17	0.16	0.16	0.16	0.18
Isoleucine	0.32	0.34	0.34	0.36	0.33	0.31	0.32	0.34	0.33	0.35	0.31	0.32
Leucine	1.10	1.18	1.13	1.24	1.11	1.06	1.11	1.16	1.12	1.21	1.03	1.04
Tyrosine	0.18	0.24	0.16	0.17	0.25	0.24	0.26	0.24	0.24	0.27	0.24	0.24
Phenylalanine	0.44	0.47	0.44	0.48	0.45	0.44	0.44	0.46	0.45	0.48	0.42	0.43
Hydroxylysine	0.01	0.00	0.01	0.01	0.01	0.01	0.02	0.02	0.02	0.02	0.01	0.02
Ornithine	0.01	0.01	0.01	0.01	0.01	0.01	0.01	0.01	0.01	0.01	0.01	0.01
Lysine*	0.24	0.28	0.26	0.28	0.28	0.27	0.28	0.28	0.27	0.27	0.26	0.28
Histidine	0.23	0.28	0.26	0.28	0.27	0.24	0.27	0.28	0.27	0.27	0.26	0.26
Arginine	0.33	0.38	0.34	0.35	0.40	0.40	0.41	0.39	0.39	0.39	0.4	0.42
Tryptophan	0.06	0.06	0.06	0.06	0.08	0.07	0.06	0.07	0.07	0.07	0.07	0.07
Total	8.45	9.17	8.78	9.3	8.91	8.58	9.04	9.16	8.98	9.41	8.58	8.78

H = Hybrid maize; V= Maize varieties; L = Landraces; M= Hybrid mixtures; DMF = Dry masa flours. *Lysine is the limiting amino acid in the evaluated corn tortillas.
